# Assessment of corn wet distillers grains fed to crossbred bulls on feeding behavior, rumen morphology, liver abscesses and blood parameters

**DOI:** 10.1371/journal.pone.0271461

**Published:** 2022-08-11

**Authors:** Maria Betânia Niehues, Laís de Aquino Tomaz, Mateus Silva Ferreira, Welder Angelo Baldassini, Luis Artur Loyola Chardulo, Ana Bárbara Sartor, Richard Vaquero Ribeiro, Luiz Antonio Fogaça, Mário de Beni Arrigoni, Cyntia Ludovico Martins, Otávio Rodrigues Machado Neto

**Affiliations:** 1 College of Veterinary Medicine and Animal Science, São Paulo State University (UNESP), Botucatu, São Paulo, Brazil; 2 School of Agricultural and Veterinarian Sciences, São Paulo State University (UNESP), Jaboticabal, São Paulo, Brazil; The University of Sydney, AUSTRALIA

## Abstract

Corn ethanol production has been growing in Brazil in the last ten years, generating by-products to feedlot diets. This study evaluates the effects of the inclusion of low-fat corn wet distillers grains (LF-WDG) on feeding behavior, ruminal health, liver abscesses and blood parameters of F1 Angus-Nellore bulls feedlot finished. Our hypothesis is that evaluation of data from feeding behavior, rumen and liver health would help to explain animal performance. In this trail, one-hundred animals were fed for 129 days with diets containing amounts of 0 (control), 15, 30 and 45% of LF-WDG replacing corn grain and soybean meal. Evaluations of fluctuation of dry matter intake (DMI) were carried out. Additionally, feeding behavior data were assessed by monitoring (24-h period) the feeding, rumination, time spent eating (TSE), and time expended on other activities (resting and number of meals per day). Blood variables such as pH, bicarbonate, total CO_2_ content, and base excess in extracellular fluid (Beecf) were determined. After slaughter, rumen epithelium was classified according to the incidence of lesions (rumenitis) and abnormalities (papillae clumped), and samples were collected for morphology and histology evaluations. Moreover, livers were scored for severity of abscesses as follow: as unabscessed (0), one or two small abscesses (A−), two to four small active abscesses (A) or one or more large, active abscesses (A+). The DMI (kg/day) differed (*P =* 0.03) among treatments and there is a tendency of 15 and 30 LF-WDG (% DM) had lower %DMI fluctuation compared to 0 or 45%. The TSE increased linearly (*P* < 0.01) as the amounts of inclusion of LF-WDG increased. Moreover, neutral detergent fiber (NDF) intake, NDF consumption rate and NDF rumination efficiency increased linearly (*P <* 0.01) in response to LF-WDG feeding. The incidence of rumenitis tended (*P =* 0.08) to be greater at 45% LF-WDG, while keratin thickness decreased linearly in bulls fed LF-WDG (*P <* 0.01). The severity of liver abscesses (score A+) increased linearly (*P* = 0.02). Regarding blood parameters, only Beecf decreased linearly (*P <* 0.01) in response to LF-WDG feeding. Therefore, the hypothesis of the current study was confirmed. We previous reported that F1 Angus-Nellore bulls fed LF-WDG show greater weight gain (1.94 ± 0.09 kg/day) and final body weight (620 ± 18.8 kg) when compare to control (1.8 ± 0.09 kg/day and 602 ± 18.8 kg, respectively). Here, we conclude that inclusion of 15 to 30% LF-WDG in feedlot diets improved feeding behavior without impairing ruminal health and blood parameters, driving performance and weigh gain of crossbred bulls. However, bulls fed 45% LF-WDG had greater severity of liver abscesses.

## Introduction

Wet distillers grains (WDG) are rich in protein and energy when compared to corn, being an excellent feed resource for feedlot cattle [1]. The WDG used in the United States of America (USA) have about 9.2% fat [2]). Recently, the ethanol industries in USA and Brazil have generated this ingredient with 5% or less of ether extract, known as low-fat WDG (LF-WDG) or de-oiled WDG. This by-product contains around 4.85% starch because most of it is converted o ethanol by the fermentation process. The regulation of feed intake in animals finished with by-products such as LF-WDG has not been fully understood in bulls. In the literature, a few studies have evaluated the effect of LF-WDG on the feeding behavior of steers [3,4]. It is well known that a higher proportion of rumen degradable starch is associated with an increase in organic acids production and considerable reduction in acetate/propionate ratio. In this context, when WDG is used to replace ground corn, proportions of distillers grains above 30% in finishing diets changes the nutritional profile of these diets due to the drastic reduction in starch contents and to the increase of crude protein content [5]. Little is known about how these nutritional strategies may affect feeding behavior cattle in feedlots.

In addition, partial replacement of starch by LF-WDG was shown to reduce the incidence of ruminal acidosis and liver abscesses [6,7]. However, inclusion of dry distillers grains with solubles (DDGS) in feedlot diets did not result in any mitigation in the prevalence of rumenitis in one study [8]. Another study reported that the inclusion of corn DDGS did not affect the incidence of liver abscesses in feedlot cattle [9]. Nevertheless, opposite results were reported in the literature [10], in which inclusion of DDGS as a replacement for barley silage promotes liver abscess in crossbred yearling steers. Thus, more studies are need to understand the effects of distillers grains on rumen health of bulls.

Furthermore, few studies evaluates the effects of increasing amounts of LF-WDG (and consequent changes in starch content of feedlot diets) on ruminal morphometry and blood parameters of F1 Angus-Nellore bulls. The concentrations of plasma metabolites (cholesterol, triglycerides, urea, albumin and creatinine) and the activities of enzymes involved in energy metabolism can be useful indicators of changes in the metabolic status of beef cattle [11]. Moreover, the digestion of starch increased propionate concentrations in the rumen, which is an important hypophagic compound in ruminants [12]. We previous reported that the inclusion of 30% LF-WDG in finishing diets of F1 Angus-Nellore improves feed intake and, consequently, final body weight, hot carcass weight, and rib eye area [13]. In the current study, our hypothesis was that evaluation of data from feeding behavior, rumen and liver health, as well as blood metabolic profile from the same animals would help to explain the of growth and performance results.

This study evaluates the effects of the inclusion of LF-WDG in feedlot diets of crossbred bulls. A better understanding of the feeding behavior, ruminal morphometry, incidence of liver abscesses and blood parameters in response to feeding LF-WDG under feedlot conditions will help guide interventions that could improve animal health, welfare and performance.

## Material and methods

### Animals, diets and experimental design

The cattle in this study were cared according to the protocol approved by the São Paulo State University animal ethics board (CEUA Protocol 0067/2017). A hundred F1 Angus-Nellore bulls with an average initial body weight of approximately 370 ± 39.45 kg and average age of 24 months were used. The animals were kept in collective pens 5×6 meters (1.0 m linear bulk per animal) with concrete floor, equipped with a shell-type water trough, with a capacity of five animals per pen. The experiment was conducted with five pens per treatment, totaling 25 animals / treatment. Initially, the animals went through a period of pre-adaptation to the facilities and feeding for six days in which they received Tifton 85 hay and soybean meal. The animals were fed with diets containing amounts of 0 (control), 15, 30 and 45% of LF-WDG (dry matter basis) replacing corn grain and soybean meal (Table 1).

**Table 1:** Composition of the experimental diets.

Diet composition	Concentration of LF-WDG (% DM)			
	0	15	30	45
Ingredients (DM basis)				
Tifton 85 hay	4.20	4.20	4.20	4.20
Sugarcane bagasse	7.10	7.10	7.10	7.10
Ground corn	74.92	65.27	52.00	38.73
Soybean meal	10.36	4.78	2.94	1.10
LF-WDG ^a^	0	15	30	45
Supplement mineral-vitamin ^b^	3.42	3.42	3.42	3.42
Potassium chloride	0	0.23	0.34	0.45
Chemical composition	Mean			
Dry matter (DM), % as fed	86.76	67.54	55.30	46.80
Ether extract (EE), % DM	3.42	3.57	3.63	3.69
Crude protein (CP), % DM	14.84	16.17	19.10	22.00
Rumen degradable protein ^c^, % CP	49.80	39.48	32.26	26.52
Rumen undegradable protein^§^, % CP	50.20	60.52	67.74	73.48
Non-fibrous carbohydrates ^d^, % DM	62.76	54.04	43.43	32.83
Starch, % DM	54.47	48.31	39.62	30.92
Neutral detergent fiber (NDF), % DM	16.23	23.46	30.83	38.21
Phisically effective NDF, % DM	9.00	9.00	9.00	9.00
Ca, % DM	0.74	0.72	0.72	0.72
P, % DM	0.36	0.41	0.48	0.55

^a^ Low-fat corn wet distillers grains.

^b^ Mineral-vitamin supplement containing: 19.5% Ca; 1.9% S; 1.5% Mg; 4.5% Na; 1.6% P; 1715 ppm Zn; 1285 ppm Mn; 428 ppm Cu; 25 ppm I; 5.7 ppm Se; 8.5 ppm Co; 286 ppm Fe; 86000 UI Vit A; 115000 UI Vit D3; 128000 UI Vit E; 32.5% Urea; 945 ppm of sodium monensin.

^c^ Estimated by the NRBC (2016) [14].

^d^ Estimated by the equation NFC = 100 - (% CP + % EE + % Ash + % NDF).

The experiment was performed in a randomized block design, and bulls were weighed at the beginning of the experiment and distributed in 2 blocks (denominated "light" and "heavy") according to the initial body weight (iBW). Light animals had an iBW ranging from 290 to 367 kg (iBW mean = 336.61 ± 18.32 kg), whereas heavy animals had an iBW ranging from 368 to 495 kg (iBW mean = 415.87 ± 31.10 kg). Then, the pens were randomly allocated among the four treatments, totaling 20 experimental units (10 pens in each block). Animals were fed for 129 d and diets were given *ad libitum* twice a day (10:00 a.m and 04:00 p.m.). One sample of the feed refusals from each pen was collected twice a week and taken to a dry-forced ventilation oven at 55°C for 72h for drying through the method 976.05 [15] to measure daily dry matter intake (DMI). The daily adjustment in dietary supply was based on the intake of the previous day in order to have 5% feed refusals.

The LF-WDG used in the current study was produced by "Front-End Fractionation" process in the ethanol industry (SJC Bioenergy, Quirinópolis, Goiás, Brazil), in which the corn germ is removed from the grain before fermentation. No condensed distillers solubles were added in the LF-WDG. For storage, the byproduct was inoculated with heterofermentative bacteria *Lactobacillus buchneri* and *Lactobacillus plantarum* (FeedtechTM Silage F600, Delaval, Tumba, Sweden) at a concentration of 1 mg/kg of LF-WDG, and were then ensiled in bag silos (5 × 60 m) at a density of approximately 1100 kg/ m^3^. Samples of LF-WDG from each bag was collected once a week to measure DM content (method 976.05).

The chemical composition of the LF-WDG supplied to the bulls during the experiment presented mean values of 32 ± 0.07% dry matter–DM (as fed), 32.8 ± 0.90% crude protein (CP), 58.9 ± 3.97% neutral detergent fiber (NDF), 37.2 ± 2.91% acid detergent fiber (ADF), 4.0 ± 0.17% ether extract (EE), 3.2 ± 0.44% ash and 1.1 ± 5.39% non-fibrous carbohydrates (NFC) (DM basis).

To minimize variations in the dry matter (DM) of the diet, the sugarcane bagasse and LF-WDG’s DM were determined and adjusted daily using the KOSTER moisture meter (Koster Moisture Tester, Koster Crop Tester, Inc., StrongsVille, OH, USA). Diets and were analyzed [15] for DM (method 976.05), CP (method 976.05, N × 6.25) and ash content (method 942.05). For NDF analysis, samples were treated with alpha amylase at a stable temperature without the addition of sodium sulfite and corrected for ash (aNDFom) [16]. The ADFom analysis was measured according to Van Soest et al. [17] and EE analysis was conducted by the Soxhlet extraction (method 920.39), following previously described procedures [15].

### Feed intake fluctuation

The evaluation of fluctuation in the DMI was carried out according to the methodology described by Bevans et al. [18]. From the daily DMI data, differences in the DMI between two consecutive days during the experimental period were calculated as follow:

$$DMI\;Fluctuation\left(\%\right)=\frac{\left|DMI_{Current\;day}-DMI_{Previous\;day}\right|}{DMI_{Previous\;day}}\times 100$$ (1)

where: DMI = dry matter intake (kg); DMID = dry matter intake of the day (kg); DMIPD = dry matter intake from the previous day (kg).

### Feeding behavior

The animals were submitted to visual observations to assess feeding behavior, which corresponded to the 29^th^, 73^rd^ and 113^th^ days of the beginning, middle and end of the finishing period, respectively, using method adapted from Robles et al [19]. Feeding behavior data were recorded by 20 trained individuals (1 per pen) every 5 min during a 24-h period for each animal as follows: time spent eating, ruminating, resting (expressed in minutes), and number of meals per day (number of visits to the trough). A meal was considered the noninterrupted time cattle stayed in the feed bunk eating the ration.

The DMI was measured on the days of data collection as the amounts of feed disappeared from the feed bunk (daily DM delivery minus daily DM as orts). The meal length in minutes was calculated by dividing time spent eating by number of meals per day. The DMI per meal in kilograms was calculated by dividing DMI by the number of meals per day. Also, time spent eating and time spent ruminating data were used to calculate the eating rate of DM (ERDM; time spent eating/DMI) and rumination rate of DM (RRDM; time spent ruminating/DMI), both expressed in minutes per kilogram of DM. Samples of diets and orts were collected for chemical analysis of NDF [17] to determine the intake of NDF on the day of feeding behavior data collection. Eating rate of NDF (ERNDF) was calculated by dividing the time spent eating by NDF intake. Rumination rate of NDF (RRNDF) was determined by dividing the time spent ruminating by NDF intake. Both ERNDF and RRNDF were expressed in minute per kilogram of NDF.

### Physically effective fiber analysis and sorting index

The evaluation of the ingredient selection index was carried out by collecting samples of the total diet and the feed refusals of the 20 pens on the days of behavioral observations. The particle size distribution was analyzed using the Penn State Particle Separator (PSPS) (Nasco, Fort Atkinson, WI, USA), determining the extent of the selection (expressed as a selection index). The PSPS was equipped with three boxes containing sieves of different diameters (19.0; 8.0; and 1.18 mm). Approximately 200 g of sample was placed on the first box (19 mm in diameter), and then the PSPS was stirred as described by Heinrichs and Kononoff [20]. Selective consumption was determined as follows: *n* intake/*n* predicted intake, in which *n* = particle fraction screens of 19 mm (long), 8 mm (medium), 1.18 mm (short), and a pan (fine). Selective consumption values equal to 1 indicate no sorting, those <1 indicate selective refusals (sorting against), and those >1 indicate preferential consumption (sorting for), as described [21].

### Blood variables

Blood samples from the jugular vein were collected after 78 and 117 days on feed. Collections were performed within three hours after feeding, in 2-milliliter syringes with the anticoagulant Heparin sodium. The following blood variables were determined with a pH/blood gas analyzer (BAYER Rapid Lab 865; Siemens Healthcare Diagnostics Inc., Deerfield, IL, USA) after sample collection as described by Brossard et al [22]: pH, bicarbonate (HCO_3_), total CO_2_ content (TCO_2_), and base excess in extracellular fluid (Beecf). Additionally, oxygen saturation (O_2_Sat) and lactate concentration was determined using a portable i-STAT^®^ 1 analyzer (Abbott I-Stat Point of Care, Princeton, NJ, USA).

The pH, pCO_2_ and pO_2_ values were corrected according to the rectal temperature of each animal, measured with a clinical thermometer. Serum blood samples were collected into evacuated tubes (BD Vacutainer, Franklin Lakes, NJ, USA) containing anticoagulant Heparin sodium, and then centrifuged at 3000 rpm for 15 minutes. The samples were then placed in labeled Eppendorf tubes and stored at −20°C until subsequent analysis for the following serum enzymes: aspartate aminotransferase (AST) and gamma-glutamyl transferase (GGT), and alanine aminotransferase (ALT). Enzyme analyses were performed colorimetrically using commercial kits (LabQuest, Campinas, SP, Brazil), and the reading of the catalytic activity was performed in a spectrophotometer (Mindray BS 120, Guangdong, China), with the temperature between 20 and 30◦C.

The contents of albumin, urea, and creatinine concentrations in the blood were determined by the colorimetric method using commercial kits (Laborlab^®^, Osasco, SP, Brazil). Cholesterol, high-density lipoprotein (HDL), and triglyceride concentrations were determined by using commercial enzymatic kits (Laborlab, Guarulhos, São Paulo, Brazil) as described by Allain et al. [23]. The concentration of very-low-density lipoprotein (VLDL) was calculated by dividing the triglyceride concentration by 5. Low-density lipoprotein (LDL) concentration was calculated as LDL = cholesterol—(HDL + VLDL).

The concentrations of blood urea nitrogen (BUN) were determined by diacetyl monoxime method [24]. Upon arrival at the laboratory, whole blood samples were analyzed for BUN concentration by an automated colorimetric procedure (Technicon AutoAnalyzer II Industrial Technicon Instruments Corp., Tarrytown, NY).

### Slaughter of animals

After 129 days on feed, bulls fed 15, 30 or 45% LF-WDG had greater final body weight when compared to the control group (602, 617, and 630 versus 615 ± 18.80 kg, respectively). The animals were transported for slaughter (approximately 500 km) in a commercial abattoir (Frigoestrela, Estrela D´oeste, São Paulo, Brazil), which is under inspection by Brazilian Federal agency, preceded by fasting of solids for at least 16 h. The animals were stunned by brain concussion using a captive dart gun, followed by bleeding, hide removal and evisceration.

### Evaluation of ruminal and hepatic health

Rumenitis was assessed after bulls were eviscerated and entire rumen contents washed. Each entire rumen surface was scored, whereby rumen epithelium was classified according to the incidence of lesions (rumenitis) and abnormalities (e.g., papillae clumped) as described by Bigham and McManus [25] using a scale of zero (no lesions and abnormalities noted) to 10 (severe ulcerative lesions). All rumens were scored by two trained individuals who were blind to the treatments, and final data represented the average of the two scores.

Livers were scored for severity of abscesses using the system reported by Brink et al. [26], whereby liver abscesses were scored as unabscessed (0), one or two small abscesses (A−), two to four small active abscesses (A) or one or more large, active abscesses (A+). A trained technician, in the slaughterhouse, performed the classification of abscesses. In addition, the percentage of animals affected by abscesses within each treatment was considered for analysis, obtaining the incidence of liver abscesses.

### Morphology and histology of the rumen papillae

Samples were collected for further morphology and histology evaluations of the ruminal epithelium, as described [27]. Briefly, to evaluate the ruminal papillae morphology, a 1-cm^2^ fragment of each rumen was collected from dorsal cranial sac (atrium ruminis) and placed into a phosphate buffer solution for future morphometric measurements according to Resende Júnior et al. [28]. These samples were immediately placed in sterile tubes (80 mL) identified with the animal number, containing 70% alcohol solution, storing them in polystyrene boxes for transport.

Manually, number of papillae (NOP) per square centimeter of rumen wall was determined; 12 papillae were randomly collected from each fragment and scanned, and mean papillae area (MPA) was determined using an image analysis system (Image Tool, version 2.01 alpha 4, UTHSCSA Dental Diagnostic Science, San Antonio, TX, USA). The rumen wall absorptive surface area (ASA), expressed in square centimeters, was calculated as follows: 1 + (NOP × MPA) − (NOP × 0.002); where 1 represents the 1 cm^2^ fragment collected and 0.002 is the estimated basal area of papillae in square centimeters, as described in previous study with Nellore cattle [29]. Papillae area expressed as percentage of ASA was calculated as follows: (NOP × MPA) / ASA × 100.

Similarly, a 1 cm^2^ fragment of each rumen was collected from the ventral cranial sac for histological assessment. Histological sections were stained with hematoxylin and eosin, embedded in paraffin wax and sectioned [30]. Histological measurements, such as papillae height, papillae width, papillae surface area, and keratinized layer thickness were performed in 4 papillae per animal using a computer-aided light microscope image analysis (Leica Qwin Image Analyzer, McBain Systems, CA, USA).

### Statistical analysis

Data were analyzed using the PROC MIXED procedure of the SAS statistical software (version 9.2; SAS Institute, Cary, NC, USA), where the pen was the experimental unit, the inclusion of LF-WDG was the fixed effect and the block, random effect. Before analysis, all data were tested for normality, evaluating the profile of its residue using PROC UNIVARIATE. Data with Shapiro-Wilk values ≥ 0.05 were considered normal. Thus, variables that did not show normality, such as: selection index, lactate and GGT, were transformed by log to achieve normal distribution. Liver abscesses data were analyzed by PROC GLIMMIX procedure of SAS.

Orthogonal contrasts were used to detect linear and quadratic effects of LF-WDG amounts using the SAS CONTRAST option. For all data, *P* ≤ 0.05 values were considered significant effects and trends were considered at 0.05 < *P* ≤ 0.10, according to the model:

$$Y_{ijk}=\mu +T_{i}+B_{j}+e_{ijk}$$ (2)

where: *Y*_*ijk*_ = observation regarding the k^th^ experimental unit (pen) of the i^th^ treatment in the j^th^ block; μ = overall mean; *T*_*i*_ = effect of the i^th^ treatment, with i = 0% of LF-WDG; 15% of LF-WDG; 30% of LF-WDG; 45% of LF-WDG; *B*_*j*_ = block effect; *e*_*ijk*_ = experimental error regarding the k^th^ experimental unit of the i^th^ treatment in the j^th^ block.

## Results

### Feed intake and weight gain

There was a tendency to a quadratic effect (*P* = 0.09) on DMI (kg/d) and the plateau was reach in the group of animals fed 15% LF-WDG (Table 2). There is a tendency of 15 and 30 LF-WDG (% DM) had lower %DMI fluctuation compared to 0 or 45%. As feed intake drives performance, average daily gain (ADG) and hot carcass weight (HCW) were affected by feedlot diets. As previous reported [13] (S1 Table), greater ADG was observed in bulls fed 15, 30 or 45% LF-WDG (1.90, 2.01, or 1.91 ± 0.09 kg/day, respectively) when compared to control (1.80 ± 0.09 kg/day). As expected, greater HCW was observed in bulls fed 15, 30 or 45% LF-WDG (340.33, 348.54, or 356.10 ± 10.48 kg, respectively) versus control diet (347.87 ± 10.48 kg).

**Table 2:** Feeding behavior of F1 Angus-Nellore bulls fed increasing amounts of low-fat corn wet distillers grains (LF-WDG).

Item ^a^	Concentration of LF-WDG (% DM)				SEM ^b^	*P-*value ^c^		
	0	15	30	45		Treatment	L	Q
DMI, kg/day	10.74a	11.53b	11.44b	11.35b	0.33	0.03	0.13	0.09
DMI, %BW	2.21	2.35	2.30	2.31	0.06	0.08	ns	ns
DMI fluctuation, %	4.31	3.45	3.96	4.41	0.30	0.09	ns	Ns
Time spent resting, min/day	1022.97	1019.55	1005.20	989.87	16.182	0.46	ns	ns
Time spent eating, min/day	158.37a	159.36a	175.47b	194.13c	8.403	0.01	<0.01	0.29
Time spent ruminating, min/day	258.28	261.45	258.80	256.40	12.512	0.99	ns	ns
Chewing time, min/day	416.65	420.81	434.27	450.53	16.592	0.47	ns	ns
NDF intake, kg	1.22a	2.18b	2.80c	3.85d	0.122	<0.01	<0.01	0.62
Number of meals per day	13.83	12.75	14.27	16.16	0.931	0.08	ns	ns
Consumption rate, min/kg								
DM	15.14a	14.26a	16.60a	17.56a	1.154	0.19	ns	ns
NDF	94.60	61.29	54.90	46.27	4.891	<0.01	<0.01	0.01
Rumination efficiency, min/kg								
DM	24.48	23.54	24.31	23.17	1.652	0.91	ns	ns
NDF	153.17d	101.35c	80.21a	60.96b	7.305	<0.01	<0.01	0.01
Average time per meal, min/day	12.18	13.75	13.00	12.19	0.732	0.58	ns	ns
DMI per meal, kg	0.87	1.07	0.82	0.75	0.101	0.17	ns	ns

^a^ DMI = dry mater intake; BW = body weight; Time spent eating and time spent ruminating data were used to calculate the eating rate of DM (ERDM; time spent eating/DMI) and rumination rate of DM (RRDM; time spent ruminating/DMI). Eating rate of NDF (ERNDF) was calculated by dividing the time spent eating by NDF intake. Rumination rate of NDF (RRNDF) was determined by dividing the time spent ruminating by NDF intake.

^b^ SEM: standard error of the mean. Each treatment consisted of five pens (5 animals/pen), totaling 20 experimental units.

^c^ Orthogonal contrasts- L: linear effect of the including amounts of LF-WDG; Q: quadratic effect of the including amounts of LF-WDG. For all data, *P* ≤ 0.05 values were considered significant effects and trends were considered at 0.05 < *P* ≤ 0.10. ns: non-significant. a-d: Means with different letters in the same row differ (*P* < 0.05).

### Feeding behavior and particle selection

The time spent eating increased linearly (*P* < 0.01) as the amounts of LF-WDG increased (Table 2). The NDF consumption rate and NDF rumination efficiency responded in a quadratic way (*P =* 0.01) to the inclusion of LF-WDG. Variables such as time spent resting, eating, ruminating, and chewing were not affected by LF-WDG feeding (*P* > 0.05). Moreover, there were no differences for average time per meal and DMI per meal (*P* > 0.05).

Regarding the particle size selectivity, there were no differences (*P* > 0.05) for long (19 mm), medium (8 mm) and short (1.8 mm) (Table 3).

**Table 3:** Selectivity of particle size by F1 Angus-Nellore cattle in feedlot fed increasing amounts of low-fat corn wet distillers grains (LF-WDG).

Particle size (mm)^a^	Concentration of LF-WDG (% DM)				SEM ^b^	*P-*value ^c^		
	0	15	30	45		Treatment	L	Q
Long	1.02	1.01	1.03	1.01	0.007	0.27	ns	ns
Medium	1.02	1.01	1.03	1.01	0.006	0.10	ns	ns
Short	1.01	1.00	1.00	1.00	0.002	0.52	ns	ns
Thin	0.98	0.99	0.99	0.99	0.004	0.21	ns	ns

^a^ >19 mm = Long; >8 mm = Medium; >1.8 mm = Short; <1.8 mm = Thin.

^b^ SEM: standard error of the mean. Each treatment consisted of five pens (5 animals/pen), totaling 20 experimental units.

^c^ Orthogonal contrats—L: linear effect for LF-WDG in the diet; Q: quadratic effect for LF-WDG in the diet. For all data, *P* ≤ 0.05 values were considered significant effects and trends were considered at 0.05 < *P* ≤ 0.10. ns: non-significant.

### Ruminal health and liver abscesses

The treatments did not influence the AAP, ANP, ASA, and the RPSA (*P* > 0.05) (Table 4). However, the incidence of rumenitis increased linearly with the concentration of LF-WDG (*P* < 0.01). Regarding the histology of the rumen papillae, no linear or quadratic effects were found for width, height, and area of the papillae (*P* > 0.05). On the other hand, as the concentration of LF-WDG increased, a quadratic effect was found for the thickness of keratin (*P* < 0.01), being lower in animals fed 45% LF-WDG.

**Table 4:** Morphology, histology and liver abscesses of F1 Angus-Nellore bulls fed increasing amounts of low-fat corn wet distillers grains (LF-WDG).

Item	Concentration of LF-WDG (% DM)				SEM ^b^	*P*-value ^c^		
	0	15	30	45		Treatment	L	Q
Morphology of the papillae								
Average area of papillae (AAP), cm²	0.65	0.74	0.60	0.69	0.061	0.19	ns	ns
Average number of papillae (ANP), n	63.45	64.29	62.77	59.82	2.620	0.59	ns	ns
Absorptive surface area (ASA), cm²	41.78	46.97	37.07	40.03	3.301	0.17	ns	ns
Representativeness of papillae in absorptive area (RPSA), %	97.37	97.99	97.30	97.48	0.213	0.12	ns	ns
Rumenitis index ^a^	1.75a	1.93b	1.76a	2.49c	0.141	<0.01	<0.01	0.08
Papillae histology								
Width, mm	0.43a	0.47a	0.39b	0.46a	0.012	<0.01	0.80	0.19
Height, mm	4.02a	4.41a	3.22b	4.02a	0.241	<0.05	0.26	0.41
Area, mm²	1.69a	2.23b	1.19a	2.00b	0.125	<0.01	0.84	0.27
Keratin thickness, µm	37.87a	29.55b	20.59c	20.15c	0.152	<0.01	<0.01	<0.01
Liver abscesses, %								
Unabscessed (0)	80.00	68.00	80.00	68.00	3.462	0.12	ns	ns
One or two small abscesses (A−)	8.00	0.00	4.00	0.00	1.914	0.24	ns	ns
Two to four small active abscesses (A)	0.00	8.00	0.00	0.00	2.001	0.36	ns	ns
One or more large, active abscesses (A+)	12.00a	24.00b	16.00a	32.00c	4.432	0.05	0.02	0.10
Incidence, %	21.00	24.00	20.00	32.00	7.011	0.26	ns	ns

^a^ Rumen epithelium was classified according to the incidence of lesions (rumenitis) and abnormalities (e.g., papillae clumped) as described by Bigham and McManus [25] using a scale of 0 (no lesions and abnormalities noted) to 10 (severe ulcerative lesions).

^b^ SEM: standard error of the mean. Each treatment consisted of five pens (5 animals/pen), totaling 20 experimental units.

^c^ Orthogonal contrats—L: linear effect for LF-WDG in the diet; Q: quadratic effect for LF-WDG in the diet. For all data, *P* ≤ 0.05 values were considered significant effects and trends were considered at 0.05 < *P* ≤ 0.10. a-c: Means with different letters in the same row differ (*P* < 0.05).

The severity of liver abscesses (A+) increased linearly (*P =* 0.05) with the concentration of LF-WDG in the diet.

### Blood parameters

The results of the blood metabolic profile were described in Table 5. The Beecf and bicarbonate decreased linearly (*P* < 0.01) with the increase in the concentration of LF-WDG in the diet (*P =* 0.002). The total CO_2_ decreased linearly (*P* < 0.01) with the increase in the concentration of LF-WDG. For the variables pH, CO_2_ pressure, O_2_ pressure, O_2_ saturation and lactate, no differences (*P* > 0.05) were observed among treatments.

**Table 5:** Blood variables of F1 Angus-Nellore bulls fed increasing amounts of low-fat corn wet distillers grains (LF-WDG).

Item ^a^	Concentration of LF-WDG (% DM)				SEM ^b^	*P*-value ^c^		
	0	15	30	45		Treatment	L	Q
pH	7.39	7.38	7.40	7.37	0.014	0.23	ns	ns
Beecf, mmol/L	2.75	2.75	1.25	0.45	0.571	<0.05	<0.01	0.38
Bicarbonate, mmol/L	27.12	27.48	25.52	25.32	0.480	<0.01	<0.01	0.48
CO_2_ pressure, mmHg	46.10	46.99	43.21	45.68	1.423	0.32	ns	ns
O_2_ pressure, mmHg	35.25	35.55	35.27	36.66	0.982	0.68	ns	ns
Total CO_2_, mmol/L	28.40a	28.80a	27.04b	26.55b	0.491	<0.05	<0.01	0.33
Saturation of O_2_, %	58.45	57.95	58.86	59.80	2.384	0.92	ns	ns
Lactate, mmol/L	2.32	1.71	1.50	2.06	0.162	0.39	ns	ns
ALT, UI/L	18.55a	15.30b	15.20b	13.70c	1.180	0.04	<0.01	0.45
AST, UI/L	79.20	82.05	97.25	91.15	6.541	0.17	ns	ns
GGT, UI/L	25.40	25.00	25.75	24.40	0.104	0.78	ns	ns
Albumin, mg/dL	3.17	3.17	3.22	3.28	0.062	0.49	ns	ns
Creatinine, mg/dL	1.34	1.26	1.23	1.17	0.071	0.25	ns	ns
Urea, mg/dL	42.20a	45.35b	48.15c	57.92d	2.101	<0.01	<0.01	0.02
BUN, mg/dL	19.71a	21.18b	22.49b	27.05c	0.982	<0.01	<0.01	0.02
Cholesterol, mg/dL	107.70a	102.55a	115.65b	124.55b	3.655	<0.01	<0.01	0.06
HDL, mg/dL	84.35a	79.55a	87.65b	93.00b	3.291	0.05	0.03	0.13
LDL, mg/dL	20.24	19.93	25.17	28.28	3.340	0.59	ns	ns
VLDL, mg/dL	3.11	3.07	2.83	3.28	0.192	0.44	ns	ns
Triglycerides, mg/dL	15.55	15.33	14.15	16.38	0.961	0.44	ns	ns

^a^ Beecf: base excess in the extracellular fluid; ALT: Alanine aminotransferase; AST: Aspartate aminotransferase; GGT: Gamma-glutamyl aminotransferase; BUN: Blood urea nitrogen; HDL: high-density lipoprotein; VLDL: very-low-density lipoprotein; LDL: low-density lipoprotein.

^b^ SEM: standard error of the mean. Each treatment consisted of five pens (5 animals/pen), totaling 20 experimental units.

^c^ Orthogonal contrats—L: linear effect for LF-WDG in the diet; Q: quadratic effect for LF-WDG in the diet. For all data, *P* ≤ 0.05 values were considered significant effects and trends were considered at 0.05 < *P* ≤ 0.10. a-c: Means with different letters in the same row differ (*P* < 0.05).

The blood concentration of the ALT enzyme decreased linearly (*P* < 0.01) with the increase concentration of LF-WDG (Table 5), while no difference for AST and GGT enzymes was observed. Moreover, there were no differences for albumin and creatinine concentrations in response to LF-WDG feeding (*P* > 0.05).

The variables urea and blood urea nitrogen increased linearly (*P <* 0.01) as the amounts of LF-WDG increased. Total cholesterol increased linearly (*P* < 0.01) with increasing concentrations of LF-WDG, being greater in bulls fed 45% LF-WDG. Regarding plasma lipoproteins, a linear increase in HDL was observed (*P <* 0.05). No treatments effects were observed on LDL and VLDL levels (*P* > 0.05).

## Discussion

### Feed intake fluctuation and feeding behavior

The hypothesis of the current study was confirmed. Our previous results [13] show that the inclusion of LF-WDG in finishing diets of F1 Angus-Nellore improves feed intake, performance, and carcass traits without impacting beef quality. The DMI observed in treatments with 15% and 30% LF-WDG agree with literature [7] including data from eight different studies. These authors reported quadratic responses for the DMI, being greater in animals fed 30% LF-WDGS. Others researchers [31,32] evaluating increasing amounts of WDG (0, 15, 30, 45 and 60%) replacing ground corn, observed similar effects on DMI (quadratic response), whereby maximum DMI occurred at concentrations of 15 and 30% LF-WDG. Moreover, the fluctuations of DMI observed in F1 Angus-Nellore bulls fed LF-WDG agree with a recent meta-analysis [33], in which differences of 1.04% in DMI fluctuation was associated with a difference in performance, feeding behavior, blood gas profile and rumenitis of zebu cattle grouped by low and high-DMI fluctuation groups.

One American study reported that decline of DMI in response to inclusion of distillers grains above 30 to 40%, can be partially explained by the high concentration of lipids in the distillers grains [34]. In the current study, the lower starch content in LF-WDG diets would help explain why DMI trend to increase in those animals, probably due to changes in the microbial populations. Additionally, we measured short chain fatty acids (SCFAs) in ruminally cannulated Nellore bulls and found that effects on DMI of animals that received LF-WDG (15, 30 or 45%) occurs due to the changes in dietary carbohydrate source and, consequently, in the ruminal environment. [35].

In addition, propionate and non-esterified fatty acids are primary fuels that cause hypophagia in ruminants, but amino acids are also extensively metabolized in the liver, especially when the diet contains excess protein, resulting in greater ammonia absorption. This implies an increase in urea synthesis and the generation of oxidizable carbon from the catabolism of amino acids in the liver for a long time, leading to greater satiety [12], which could also explain the tendency to reduction in feed intake at LF-WDG amounts above 30%.

Bulls from LF-WDG treatment needed to spend more time feeding to consume 1 kg of DM compared to control ones, resulting in an increased time spent eating, which trend to being greater at 45% LF-WDG. Researchers reported an increase in feeding time when WDG was included at 40% compared to 0% in dry-rolled corn-based diets [3]. However, these authors did not observe any effect of the WDG inclusion for meals, average time per meal or DMI per meal.

The tendency in increased meals observed in the present study also may be due to differences in fiber content in feedlot diets (control versus LF-WDG) and might be associated with changes in the rumen environment. Increasing the frequency and distribution of meals throughout the day can help control rumen pH since it leads to better synchronization in the time between SCFA, saliva production (if rumination occurs between meals) and absorption and passage of rumen organic acids [36]. However, researchers observed that effects of meal frequency and duration on ruminal pH were small, while daily DMI had a large effect on ruminal pH in Australian beef cattle fed commercial diets [37].

The fiber content present in the LF-WDG was not effective in stimulating rumination activity and, consequently, the production of saliva to assist in ruminal buffering was probably limited [38]. On the other hand, the LF-WDG contributes to the total NDF of the diet, reflecting in the increase of the NDF intake. This was expected since the NDF content of the LF-WDG (56.50%) is much higher than the concentration present in corn (around 8%). In the literature, studies have reported increased NDF intake with increased WDG in the diet [9,31,39].

### Rumen health and liver abscesses

The papillae increase the ASA of the rumen, contributing deeply to a greater absorption surface / cm^2^ of rumen wall and ANP for SCFA absorption. The inclusion of LF-WDG affected the rate of rumenitis in the evaluation of the morphology of the ruminal epithelium, being higher in animals fed 45% LF-WDG. This result may be explained by the greater selection of concentrate ingredients (particles smaller than 1.8 mm), suggesting that F1 Angus-Nellore bulls had more preference for the intake of small particles. Opposite results were reported by researchers [27] evaluating Nellore bulls fed high concentrate diets, whereby the number of papillae, mean papillae area, papillae area as a percentage of absorptive surface, and rumenitis incidence were similar. Differences among studies on rumen papillae variables may be due to chemical composition of the experimental diets, specially starch amounts and NDF content.

The animals fed LF-WDG had a higher rate of rumenitis, resulting in greater severity of liver abscesses. Similarly, researchers [31,32] evaluating WDG amounts of 15% and 30% replacing corn, containing 7.5% alfalfa hay as a source of forage, reported that the incidence of liver abscesses tended to increase linearly as the concentration of WDG in the diet increased. The effects on incidence of liver abscesses may be due the excess protein content of LF-WDG used as energy, which is deaminated in the liver to produce ketone bodies, and urea is excreted. Moreover, the higher protein content of LF-WDG diets can generate higher ammonia levels in the liver, further affecting the ability of the liver to detoxify lipopolysaccharides and, consequently, increasing liver abscess formation. However, the incidence of liver abscess was not influenced by the level of inclusion of distillers grains in other studies [9,40].

### Blood parameters

The blood metabolic profile indicated lower blood buffering in animals fed LF-WDG, considering the concentrations of Beecf, bicarbonate and total CO_2_. Although blood pH was similar for all treatments, which may indicate that the cattle did not present metabolic acidosis [41], urine pH is much more responsive to the animals acid/base balance and would be a better indicator for potential metabolic acidosis in future studies. The lower concentrations of Beecf and bicarbonate in the blood can be explained by the higher DMI of animals fed LF-WDG, associated with high fermentability of the fiber and the same concentration of peNDF, resulting in reduced rumination time.

The decrease in serum concentrations of the ALT enzyme with the increase in LF-WDG concentrations in the diet can be explained by the lower propionate supply for hepatic gluconeogenesis in diets containing LF-WDG. Thus, this might led to greater use of the glycogenic amino acid alanine for the glucose formation. This enzyme is mainly distribuited in liver, kidney, skeletal muscle and myocardium, in which ALT catalyzes the transfer of α-amino groups from alanine to the α-keto group of ketoglutaric acid, forming pyruvic acid, an important contributor to the citric acid cycle [42]. Therefore, greater activity of the ALT enzyme may have occurred in the liver of animals that received LF-WDG, which led to a decrease in their activity in the blood. One study reported that the progressive concentrations of ALT in the serum of rats fed increasing amounts of sorghum byproduct is indicative of increased catabolism of amino acids in the liver [43].

When included in amounts above 30%, LF-WDG causes greater production of urea and consequently greater excretion of nitrogen as uric acid, which has an associated energy cost [14]. The energy cost of ureogenesis and increase in urea excretion could impair animal performance, however, in our previous results [13], the inclusion of LF-WDG in finishing diets of F1 Angus-Nellore improves feed intake, performance, and carcass weights. Some studies reported that between 2.5% to 5% of whole body oxygen consumption was attributable to ureagenesis in the liver [44], which may relates to ALT activity observed in the current study. However, previous studies reported that tissues can adapt to changes in diet by altering tissue mass or metabolism per gram of tissue [45,46].

The higher CP in the diets can explain the increase in BUN concentrations in the blood of animals fed LF-WDG. Moreover, the renal responses that have been described with the feeding of low protein diets include decreased renal plasma flow and glomerular filtration rate (GFR) [47]. However, in other studies GFR was not significantly related to N intakes in lambs [48] and Holstein cows [49].

## Conclusions

The inclusion of LF-WDG improves DMI, feeding behavior, ruminal health and blood metabolite profile of F1 Angus-Nellore cattle feedlot finished. Overall, we conclude that inclusion of 15 to 30% LF-WDG is the best recommendation to replace ground corn as it increased the DMI and did not affect the incidence of rumenitis and preserve ruminal health. However, animals fed 45% LF-WDG had greater severity of liver abscesses.

## Supporting information

S1 Table Performance and carcass weights of F1 Angus-Nellore bulls fed increasing amounts of low-fat corn wet distillers grains (LF-WDG). ^a^ IBW = initial body weight; DMI = dry mater intake; ADG = average daily gain; FBW = final body weight; HCW = hot carcass weight; CCW = cold carcass weight. ^b^ SEM: standard error of the mean. Each treatment consisted of five pens (5 animals/pen), totaling 20 experimental units. ^c^ Orthogonal contrasts- L: linear effect of the including amounts of LF-WDG; Q: quadratic effect of the including amounts of LF-WDG. For all data, *P* ≤ 0.05 values were considered significant effects and trends were considered at 0.05 < *P* ≤ 0.10. ns: non-significant. a-c: Means with different letters in the same row differ (*P* < 0.05).
(DOCX)
