# Supplementary material for: Assessment of corn wet distillers grains fed to crossbred bulls on feeding behavior, rumen morphology, liver abscesses and blood parameters
Source: PLoS One. 2022 Aug 11;17(8):e0271461. doi: 10.1371/journal.pone.0271461 (PMC9371291; doi:10.1371/journal.pone.0271461)
Supplement: S1 Table — a IBW = initial body weight; DMI = dry mater intake; ADG = average daily gain; FBW = final body weight; HCW = hot carcass weight; CCW = cold carcass weight. b SEM: standard error of the mean. Each treatment consisted of five pens (5 animals/pen), totaling 20 experimental units. c Orthogonal contrasts- L: linear effect of the including amounts of LF-WDG; Q: quadratic effect of the including amounts of LF-WDG. For all data, P ≤ 0.05 values were considered significant effects and trends were considered at 0.05 < P ≤ 0.10. ns: non-significant. a-c: Means with different letters in the same row differ (P < 0.05). (DOCX) [file pone.0271461.s001.docx]

Table S1. Performance and carcass weights of F1 Angus-Nellore bulls fed increasing amounts of low-fat corn wet distillers grains (LF-WDG).

| Item ^a^ | Concentration of LF-WDG (% DM) | | | | SEM ^b^ | *P-*value ^c^ | | |
| --- | --- | --- | --- | --- | --- | --- | --- | --- |
|  | 0 | 15 | 30 | 45 |  | Treatment | L | Q |
| IBW, kg | 369 | 371 | 370 | 369 | 22.84 | 0.43 | ns | ns |
| DMI, kg/day | 10.74a | 11.53b | 11.44b | 11.35b | 0.33 | 0.03 | 0.13 | 0.09 |
| ADG, kg/day | 1.80 | 1.90 | 2.01 | 1.91 | 0.09 | 0.06 | 0.12 | 0.10 |
| FBW, kg | 602a | 617b | 630b | 615b | 18.80 | 0.04 | 0.12 | 0.06 |
| HCW, kg | 340.33a | 348.54b | 356.10b | 347.87b | 10.48 | 0.04 | 0.12 | 0.06 |
| CCW, kg | 326.29 | 334.26 | 341.61 | 333.62 | 10.18 | 0.08 | ns | ns |

^a^ IBW = initial body weight; DMI = dry mater intake; ADG = average daily gain; FBW = final body weight; HCW = hot carcass weight; CCW = cold carcass weight.

^b^ SEM: standard error of the mean. Each treatment consisted of five pens (5 animals/pen), totaling 20 experimental units.

^c^ Orthogonal contrasts- L: linear effect of the including amounts of LF-WDG; Q: quadratic effect of the including amounts of LF-WDG. For all data, *P* ≤ 0.05 values were considered significant effects and trends were considered at 0.05 < *P* ≤ 0.10. ns: non-significant. a-c: Means with different letters in the same row differ (*P*< 0.05).
